# Autophagy modulation in breast cancer utilizing nanomaterials and nanoparticles

**DOI:** 10.3389/fonc.2023.1150492

**Published:** 2023-05-05

**Authors:** Azar Gharoonpour, Dorsa Simiyari, Ali Yousefzadeh, Fatemeh Badragheh, Marveh Rahmati

**Affiliations:** Cancer Biology Research Center, Cancer Institute, Tehran University of Medical Sciences, Tehran, Iran

**Keywords:** autophagy, breast cancer, drug resistance, nanomaterial, nanoparticles

## Abstract

Autophagy regenerates cellular nutrients, recycles metabolites, and maintains hemostasis through multistep signaling pathways, in conjunction with lysosomal degradation mechanisms. In tumor cells, autophagy has been shown to play a dual role as both tumor suppressor and tumor promoter, leading to the discovery of new therapeutic strategies for cancer. Therefore, regulation of autophagy is essential during cancer progression. In this regard, the use of nanoparticles (NPs) is a promising technique in the clinic to modulate autophagy pathways. Here, we summarized the importance of breast cancer worldwide, and we discussed its classification, current treatment strategies, and the strengths and weaknesses of available treatments. We have also described the application of NPs and nanocarriers (NCs) in breast cancer treatment and their capability to modulate autophagy. Then the advantages and disadvantaged of NPs in cancer therapy along with future applications will be disscussed. The purpose of this review is to provide up-to-date information on NPs used in breast cancer treatment and their impacts on autophagy pathways for researchers.

## 1 Introduction

Despite the advances in breast cancer diagnosis and treatment, the incidence and mortality rates of breast cancer are still increasing especially in poorly developed regions of the world (1). The results of many studies represent the fact that a decline in infection-associated cancers is offset by the rising number of cancer cases being highly related to dietary, hormonal, and reproductive factors in countries with fast transitions in economic and communal issues. Hence, fundamental strategies for prevention, diagnosis in early stages, and novel targeted therapies can result in a decrease in expected cancer incidence (2). Most breast cancer survivors have encountered aggressive relapse. It is mainly explained by the fact that breast cancer tumors are highly heterogeneous and difficult to target (3). In anticancer therapies, the primary objective is the specific inhibition of the malignant function of cancerous cells while unaffected cells remain healthy. Ordinary treatments for cancers are encountered with inevitable side effects and drug resistance, which are discussed in the body of this article (4). In this regard, an intracellular mechanism such as autophagy targeting may be a good approach for breast cancer-targeted therapy. Autophagy as a conserved intracellular mechanism prepares the primary materials for the synthesis of vital macromolecules such as proteins by removing the unwanted molecules or organelles. Furthermore, autophagy preserves normal cells from intrinsic and extrinsic stressors promoting DNA mutations and instability that mainly lead to pre-neoplastic changes and propagation (3). Deficiency in autophagy function has led to diseases such as different malignancies. In cancer cells, autophagy has a dual role in homeostasis maintenance as a tumor suppressor in early stages and a tumor promoter at advanced stages. This important function of autophagy in cancer has led to more investigations in the field of targeting autophagy, which both inhibits and induces it in different cancers with different characteristics being critical. Many investigations in mammary cancer models revealed that the inhibition of autophagy genes can weaken the initial growth in tumors while paradoxically can result in overt metastasis and outgrowth in cancerous cells. In breast cancer, the genetic inhibition of autophagy in both early and late stages leads to the spread of tumor cells, which offers differentiation in pro-metastatic basal epithelial cells (5). In cancer initiation of mammary glands, the autophagy-related genes confer a suppressive role; however, this function is lost throughout breast cancer development, and impaired autophagy results in cancer progression (3). Of note, autophagy is detected as one of the main causes of chemotherapy resistance in breast cancer patients in a way that the inhibition of autophagy dramatically enhances the sensitivity of cancer cells to anticancer medicines (6). However, autophagy inducers could be effective in such situations and push the cells to apoptosis. Generally, efficient and genuine progress in this treatable disorder is not achieved unless a persistent and interconnected effort toward innovative procedures in cancer treatment (1). Most of the autophagy modulators that are currently available have low specificity, as they do not preferentially target a single cell type. Nanoparticles (NPs) can improve the efficacy of drugs through their high power of permeability and reduce their toxicity due to nanosized properties. They lead to more effective targeting of tissue, cells, or organelles and enhance the pharmaceutical properties of drugs such as stability, solubility, plasma half-life, and tumor accumulation. The connection between NPs and their impact on autophagy has shown that they can impact autophagy through both its induction and inhibition (7). In this review article, we aim to describe the application of different NPs and nanocarriers (NCs) in breast cancer treatment and their capability to modulate autophagy. We then discuss the advantages and disadvantages of NPs in cancer therapy, along with their future applications. The purpose of this review is to provide researchers with up-to-date information on NPs used in breast cancer treatment and their impacts on autophagy pathways.

## 2 Breast cancer

Breast cancer is one of the main causes of death in most parts of the world, especially in low-income areas (1). The incidence of breast cancer has increased in Western countries from the 1980s to 1990s and then has been stable due to better detection and the use of modern treatment. In 2020, almost 2.3 million women were diagnosed with breast cancer worldwide, and approximately 700,000 individuals died (8). According to the World Health Organization (WHO) prediction, by 2040, newly diagnosed breast cancer cases are expected to rise by almost 40% every year. A dramatic increase in the trend of breast cancer will be observed especially in countries with a low human development index (HDI), where the number of new cases and the mortality rate is projected to be doubled by 2040 (9). In Iran, breast cancer was determined to have the highest incidence rate per 100,000 women and the second-highest mortality rate after colorectal cancer in 2020 (10).

The risk factors for breast cancer are categorized as reproductive and non-reproductive factors, based on economic issues. Major reproductive factors include age, age of menarche, age at first pregnancy, some indicators of ovarian activity, age at natural menopause, duration of breastfeeding, history of breast disease, genetic status, nulliparity, and familial history of breast cancer (11). However, non-reproductive risk factors comprise obesity, lifestyle, being overweight during a post-menopausal state, and drinking level (2, 12). In Iran, other factors such as hormone replacement therapy, passive smoking, advanced maternal age at pregnancy, abortion, high levels of sugar consumption, and the genotype of Arg/Arg could also increase the risk of breast cancer development, whereas late menarche, breastfeeding for at least 13–24 months, routine exercise, and having vegetables in diet reduce the risk of breast cancer. The relationship between the polymorphism in codon 72 of the p53 gene and the risk of breast cancer shows that although the genotype of Arg/Pro was not related to the development of breast cancer, the genotype of Arg/Arg raised the risk of breast cancer significantly (13).

### 2.1 Breast cancer staging

In the 1940s and 1950s, the first staging of breast cancer was reported by Pierre De-noix, which was mainly based on the anatomic shape of the breast (14, 15). Lately, an extensively detailed system of staging cancers has been developed by the American Joint Committee on Cancer (AJCC) and has been updated eight times. In the breast cancer chapter, levels of estrogen receptor (ER) and progesterone receptor (PR) expression, human epidermal growth factor receptor 2 (HER2) or erythroblastic oncogene B (ERBB2) expression, histologic grade, regional lymph node involvement, distant metastases, and prognostic biomarkers are included in order to confer precise prognosis and guide treatment decisions (14, 16). This system is named TNM staging, which shows the status of tumors (T), lymph node involvement (N), and the level of tumor metastasis (M).

The combination of immune histological data such as the tumor grade, the determination of hormone receptor status, and a multi-gene panel with anatomic information results in a more explicit prognosis. However, tumors with lower-graded ER− and PR− tend to be more common among populations; therefore, multi-gene panels should be performed for further information (17).

### 2.2 Biomarkers in breast cancer

The biomarkers can be extremely helpful in the determination of breast cancer, especially in the early stage, to achieve a better prescription. In general, they are classified into tissue, genetic, and serum markers. The tissue markers or hormone receptors, namely, ER and PR, are critical for identifying patients that should be treated with hormone therapy. ER and PR should be tested for all patients with a breast cancer diagnosis. Nevertheless, ER alone is a weak prognostic factor (18). HER2 measurement is essential for all patients with invasive breast cancer and is the indicator for the patients who may be treated with trastuzumab and who may benefit from anthracycline-based adjuvant chemotherapy (19).

The most important genetic markers of breast cancer are defined as breast cancer gene 1 (BRCA1) and breast cancer gene 2 (BRCA2) mutation genes, which are the most powerful predictive tools to identify patients suffering from breast and ovarian cancer with a 40%–80% risk of developing cancer (20). In primary tumors, DNA ploidy is found to be an independent prognostic tool for surgical cases. Aneuploidy implies genetic abnormalities like single-nucleotide point mutations and changes in chromosome structure. Diploid tumors have a slightly longer survival time than those diagnosed with aneuploid tumors (21). The use of circulating tumor DNA (ctDNA), as a representative of tumorous DNA in the plasma or other body fluids of patients with cancer, is also important in detecting tumor cells. Detecting ctDNA reduces the need for tumor biopsy and helps a physician to determine the effective treatment, and a decrease in the levels of ctDNA in the process of treatment indicates a successful therapy (22).

The serum biomarkers are of great significance in breast cancer management therapy protocol, as they may help determine early diagnosis, prognosis, and response to therapies. They consist of carcinoembryonic antigen (CEA), the MUC-1 family agents (CA15-3, BR 27.29, MCA, and CA549), the serum cytokeratins [e.g., tissue polypeptide antigen (TPA) and tissue polypeptide-specific antigen (TPS)], and the serum oncoproteins (e.g., HER2/c-erbB-2). The approved serum markers designed for breast cancer are CA15-3 and CEA. Increased levels of CA15-3 (e.g., to 150 U/ml) and/or CEA (e.g., to 120 ng/ml) in a patient indicate a non-metastatic tumor (23). The measurement of two parameters such as urokinase-type plasminogen activator (UPA) and plasminogen activator inhibitor (PAI-1) is mostly performed in lymph node-negative patients, for whom adjuvant chemotherapy would not be helpful. Low levels of both the UPA and PAI-1 factors in such patients show a low risk of disease relapse (24).

### 2.3 Breast cancer treatment

According to the classification of breast cancer into ER and PR expression and HER2/ERBB2 gene amplification, different treatment strategies are applied (25). For ER- or PR-positive breast cancers, the first choice is the use of endocrine agents to downregulate ER signaling. For those with HER2 or HER2/neu positive, ERBB2-targeted therapy is suggested, including anti-ERBB2 antibodies (e.g., trastuzumab and pertuzumab) and small-molecule tyrosine kinase inhibitors (such as lapatinib and neratinib) (26). The main purpose of therapy for non-metastatic breast cancers is the removal of all the tumors or axillary lymph nodes by surgery, followed by postoperative radiation. Systemic therapy can also be performed preoperatively (neoadjuvant), postoperatively (adjuvant), or both. For metastatic breast cancers, the same basic categories of systemic therapy are used as neoadjuvant/adjuvant approaches (27).

**Figure 1:**
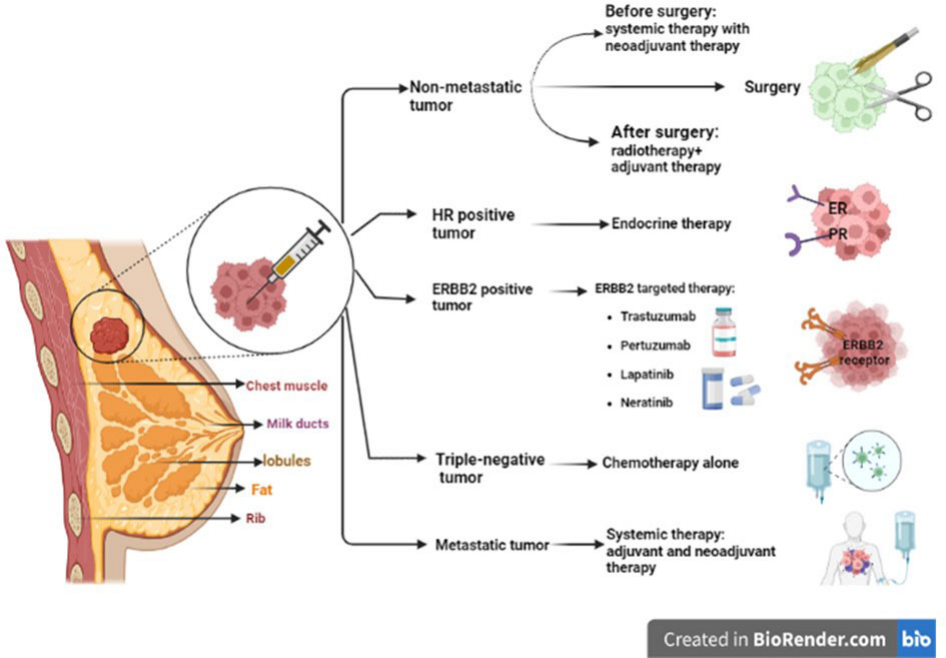
Different strategies to treat different types of breast cancer. ER/PR-positive breast cancers are much more likely to respond to hormone therapy. For HER2 or HER2/neu-positive patients, targeted therapy including anti-ERBB2 antibodies (trastuzumab and pertuzumab) and small-molecule tyrosine kinase inhibitors (such as lapatinib and neratinib) are used. For non-metastatic breast cancer, complete removal of the tumor or axillary lymph nodes by surgery and postoperative radiation is recommended. For metastatic breast cancer, systemic therapy is prescribed, such as neoadjuvant/adjuvant therapies. ER, estrogen receptor; PR, progesterone receptor; HER2, human epidermal growth factor receptor 2; ERBB2, erythroblastic oncogene B.

For patients undergoing refractory metastatic breast cancer with a diagnosed deletion in BRCA1/2 genes, treatment with poly adenosine diphosphate-ribose polymerase (PARP) inhibitors like olaparib and talazoparib is approved by the US Food and Drug Administration (FDA) (28). Conventional chemotherapy for breast cancer targets the cells non-selectively and inhibits the proliferation of both normal and cancerous cells. Therefore, gastrointestinal problems and hair loss are some of their important side effects. The other major problem is drug resistance (4), which is discussed in the following paragraph. **Figure 1** illustrates the treatment protocol for different breast cancers.

### 2.4 Breast cancer and drug resistance

Multidrug resistance (MDR) is the main reason for the relapse of breast cancer. The development of MDR is due to various molecular mechanisms, including increased expression of efflux transporters, epithelial-to-mesenchymal transition (EMT), and stem cell drug resistance (29). Cancer cells prevent the accumulation of chemical drugs inside the tumor and hinder their effectiveness *via* different mechanisms. Drug efflux transporters or efflux pumps are defensive mechanisms of cancer cells that direct anticancer drugs out of tumors. They are members of the ATP-binding cassette (ABC) superfamily involved in transporting substances into and out of cells with ATP hydrolysis (29).

Another mechanism is defined as EMT, a process by which epithelial cells lose their intercellular adhesion and apical-basal polarity properties and migrate into the mesenchyme. These cells gain new features that lead to increased drug efflux and resistance to apoptosis (30).

Breast cancer stem cells (BCSCs) are one of the major problems in drug resistance, metastasis, and cancer recurrence. Due to properties such as self-renewal, long lifespan, slow proliferation, high drug transport capacity, DNA repair mechanisms, and anti-apoptotic properties, BCSCs show intrinsic resistance to conventional therapies, and their removal is considered to increase drug susceptibility. One reasonable approach is targeting self-renewal pathways, and the other plan attacks the BCSC microenvironment. At present, some novel techniques are being discovered, including the use of cyclin-dependent kinase (CDK) inhibitors, factors that induce stem cell differentiation, and targeted immunotherapies (31).

Cancer stem cells (CSCs) also play a role in the induction of the mitochondrial-expressed genes involved in oxidative stress response, tumor survival, and drug resistance (32).

Under stressful conditions, such as reactive oxygen species (ROS) production or nutrient starvation, mitochondria undergo membrane potential depolarization and sequester into autophagosomes, under the process named mitophagy. During severe and prolonged stress, mitophagy is suppressed, and cell death pathways are activated (33). Indeed, mitophagy is a cytoprotective process during tumor progression and has a critical role in drug resistance and maintenance of stemness and self-renewal of CSCs. However, reports point to the role of mitophagy as a cellular tumor suppressor. Thus, both induction and inhibition of mitophagy contribute to the drug sensitivity of tumor cells (34). Another cause of MDR is intracellular mechanisms such as autophagy activation, discussed later in detail, which can potentially be modulated in cancer therapy.

## 3 Autophagy

Autophagy is defined as a “self-degradative” pathway that manages cellular homeostasis to provide precursors such as amino acids for the assembly of vital cellular components *via* catabolic pathways (35). In other words, autophagy helps the cells eliminate the malfunctioned organelles or macromolecules and then return the primary building materials to the manufacturing cycles of the cell, to reestablish cellular homeostasis. Autophagy is induced by various cellular stresses such as hypoxia, starvation, and infection (36). This phenomenon was indicated with studies in yeast in the 1990s by detecting autophagy-related genes (ATGs). Autophagy is classified into three types: microautophagy, macroautophagy, and chaperone-mediated autophagy (CMA) (37). Although all three types take the misfolded proteins to the lysosome, their mechanisms and morphologies are different. In microautophagy, cytoplasmic materials are directly absorbed into the lysosomal lumen through the invagination of the lysosomal membrane (38). In CMA, soluble proteins in the cytosol are selectively recognized by a 70-kDa heat shock protein (hsp70), unfolded, and translocated to lysosomes. These cytoplasmic proteins have special recognition motifs called pentapeptides. The CMA targeting motif is recognized as KFERQ and varies at different residues. For example, at “K” and “R” positions, up to two positively charged amino acids [e.g., arginine (R) or lysine (K)]; at position “F”, two hydrophobic residues [e.g., isoleucine (I), leucine (L), phenylalanine (F), or valine (V)]; and at “E” position, single negatively charged [e.g., glutamate (E) or aspartate (D)] can be placed. The “Q” can be at the N- or C-terminus of the pentapeptide and is flexible for negatively or positively charged or hydrophobic amino acids. This variation of proteins exists in approximately one-third of all the soluble cytosolic proteins. This protein–chaperone complex binds to a receptor of the lysosome and lysosome-associated membrane protein type 2A (LAMP-2A), and the substrate is released. Then, the unwanted proteins are degraded by lysosomal enzymes and reused in the next protein synthesis process (39).

Macroautophagy initiates with special double-membrane vesicles, known as autophagosomes, that progressively isolate autophagic cargo and deliver them to lysosomes by membrane fusion. An organelle that results from the fusion of an autophagosome and a lysosome is often referred to as the autophagolysosome. The digestion of cargo prepares nutrients for cell survival. Macroautophagy is directly related to autophagy (40) and is mainly considered a non-selective or general process. Nevertheless, it is noteworthy that in many cases the selective autophagy pathway has been also observed, which turns out to be essential only for cellular health. Deficiency in autophagy function can lead to diseases such as susceptibility to infections and inflammation, metabolic disorders, cardiovascular and liver problems, cancer, neurodegeneration, and acute brain damage. In tumorigenicity, autophagy performs a two-faced role of tumor suppressor and pre-oncogene (5). Because of these dual roles, finding a new targeted therapy for both activating and inhibiting autophagy pathways is under investigation (38) and will be discussed in the following sections.

### 3.1 Autophagy machinery

Autophagy contains complex steps that lead to the generation of autophagosomes and their fusion into lysosomes. The process includes several phases, namely, initiation, elongation, lysosomal fusion, and degradation, which are mediated by ATGs (3).

#### 3.1.1 Induction and initiation

In mammalian cells, the autophagy is induced by unc-51-like kinase family (ULK1/ULK2) complex (homolog of the Atg1 complex in yeast), ATG13, and RB1-inducible coiled-coil 1 (RB1CC1/FIP200). Then, C12orf44/ATG101 protein binds directly to ATG13, which occurs in mammals and not in yeast. ULK1 complex induces membrane formation in the phagophore assembly site (PAS). Accordingly, ATG2 transfers phospholipids from the endoplasmic reticulum, Golgi, and mitochondria membrane to PAS, and ATG9 transfers them to the luminal layer of the autophagosome membrane (41). As the phagophore expands, the membrane wraps around the substrate and forms a spherical autophagosome.

Autophagy is immensely regulated by two main kinases, including the mammalian target of rapamycin complex 1 (mTORC1) and AMP-activated protein kinase (AMPK), which affect ULK1 complex formation (42). mTORC1 associates with the ULK1/ULK2 complex in autophagosome under rich nutrients and inactivates the complex by its phosphorylation. However, under rapamycin treatment or starvation, mTORC1 dissociates from the complex and induces autophagy. In other words, in the abundance of amino acids and nutrients, mTORC1 inhibits autophagy through the phosphorylation of ULK1 and ATG13 (43). However, the AMPK (the autophagy inducer) can sense the AMP : ATP ratio caused by energy deprivation and be activated to start autophagy by phosphorylating several sites in the central intrinsically disordered region (IDR) in ULK1. Furthermore, the AMPK is an indirect inducer of autophagy by phosphorylation of the regulatory-associated protein of mTOR (RAP-TOR), which leads to mTOR inhibition (44).

#### 3.1.2 Nucleation

In this step, ATG14-containing class III phosphatidylinositol 3-kinase (PtdIns3K) complex binds to the autophagosome. The PtdIns3K generates the PtdIns3P complex, which is involved in the nucleation of the phagophore and consists of PIK3C3/VPS34, PIK3R4/p150, and BECN1. Some reports suggest the association of this complex with ATG14 and UVRAG is necessary for autophagosome formation. Regulation of the PtdIns3K complex is mediated through the proteins that interact with BECN1 such as BCL2. As the BCL2 binds to BECN1, it inhibits the interaction of BECN1 with PIK3C3 and inactivates autophagy (45).

#### 3.1.3 Elongation

Phagophore expands with the formation of the ATG5, ATG12, and ATG16L1 complex. As the phagophore is completed, it dissociates from the complex. The ATG8/LC3 system is also involved in the phagophore expansion. In mammals, there are several Atg8-like proteins that are the LC3 and GABARAP subfamilies. The LC3 forms LC3-1 by ATG4 and its phosphatidylethanolamine (PE)-conjugated or lipidation form, called LC3-II. The form of LC3-II is activated under starvation or other autophagy stressors. ATG9 is a transmembrane protein involved in phagophore expansion and membrane recruitment (46).

#### 3.1.4 Autophagosome fusion with lysosome

The structure of autolysosome is formed by the fusion of the outer membrane of the mature autophagosome with the lysosome (47). There are three main machinery protein families playing a crucial role in the regulation of the fusion process, including membrane-tethering factors (such as HOPS and EPG5), soluble *N*-ethylmaleimide-sensitive factor attachment proteins (SNAREs), and Rab GTPases, which are situated on the membrane. They hire the tethering complex to bridge to the facing lipid bilayer; therefore, the tethering complex employs SNARE proteins and promotes them to move autophagosomes toward the lysosome and facilitate the fusion process (48). Recently, a growing number of studies have conferred the great role of ATG8 family members in driving autophagosomes near the lysosome. In addition, the role of these members has been suggested as probable hubs in the final fusion stages. In mammals, amphisomes emerge if the autophagosome fuses with the endosome before reaching the lysosome. Microtubules are also involved in driving the autophagosomes to the lysosomes (49). Being able to assist with the UVRAG PtdIns3K complex, the UVRAG activates GTPase RAB7, which promotes autophagosome–lysosome fusion (50). Recent studies have suggested other components of the SNARE machinery system that play role in the fusion process like VAM9 and VAM7 (51).

One of the mechanisms that macroautophagy uses to selectively identify cargo is ubiquitination. The ubiquitin-binding protein SQSTM1/p62 targets the ubiquitinated proteins and then interacts with LC3-II to clear these aggregate proteins from the cytosol and move them to the lysosome (52).

#### 3.1.5 Degradation

**Figure 2:**
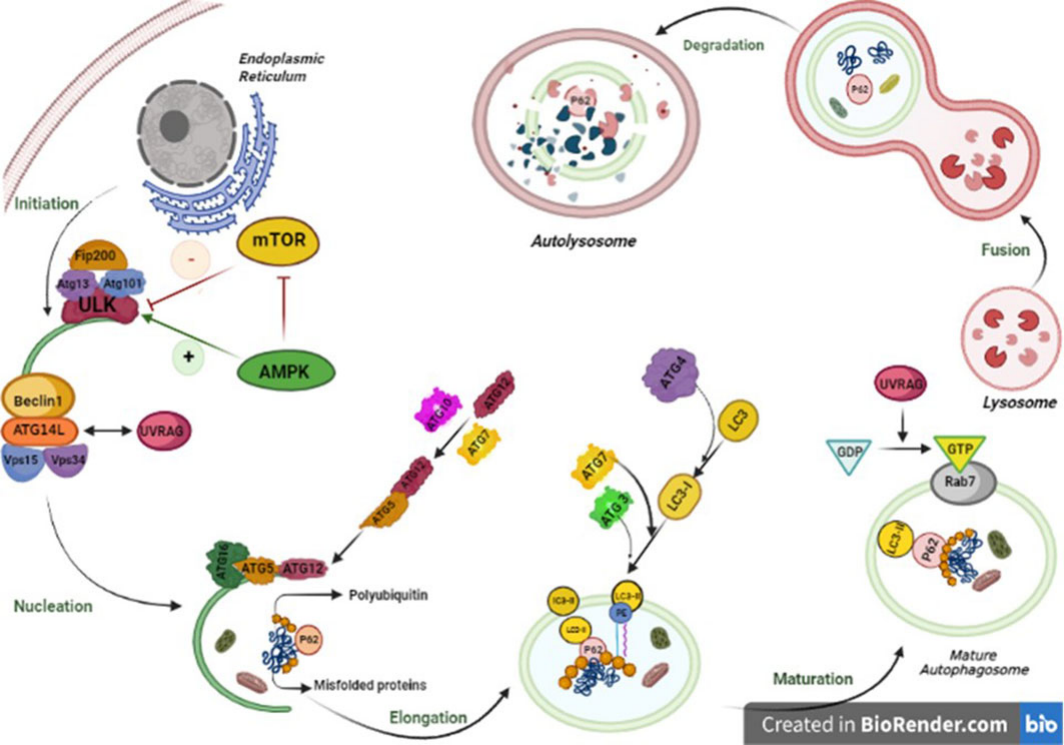
Autophagy pathway is performed in five steps from initiation to fusion with lysosome. In mammalian cells, the initiation phase of autophagy consists of autophagosome formation, which is significantly dependent on the stable complex known as ULK1-Atg13-FIP200-Atg101. The activation of the ULK1 kinase launches the activity of Beclin1 (BECN1)–VPS34 complex including BECN1, VPS34, and Beclin1 regulator 1 (AMBRA-1). In the elongation phase, the WIPI2B scaffold binds to PI3P. The mentioned complex is highly essential for employing two main proteins, ATG7 and ATG10, that can couple ATG5 to ATG12, which makes a complex with ATG16L. The Atg12–Atg5–Atg16 complex positions on the outer membrane due to ATG 5 available binding sites. The next step is the fusion of autophagosome–lysosome and includes two main phases. First, the autophagosome migration to lysosomes, which is implemented by the cytoskeleton in eukaryotic cells by Rab7 and guanosine triphosphate (GTP)-binding protein to microtubules, and second, the fusion of lysosomes, with a single bilayer membrane, and mature autophagosomes, with two lipid bilayer membranes. The last step is degradation of all components in the lysosome. The lysosomal enzymes degrade autophagic cargo.

Once the autophagosome formation is completed, its outer membrane merges with the lysosomal membrane; then, the inner layer and cargo will be degraded by hydrolases and subsequently with permeases to be competent for cell biosynthetic processes and the generation of energy (53). The product of autophagosome and the lysosome fusion is called “autophagolysosome” or “outolysosome”. The heterophagic (no-self) materials are needed in autolysosomes since approximately all lysosomes take constant flow made by endocytic pathway (54). All the autophagy steps are shown in **Figure 2**.

### 3.2 Autophagy and cancer

Autophagy has two completely different behaviors in cancer, depending on the severity of cellular stress and the state of the immune system. During tumor initiation, autophagy prevents carcinogenesis, and in the advanced stages of tumors, it aids cancer progression. In fact, autophagy acts as a tumor suppressor in the early stages of carcinogenesis by maintaining genomic stability and paradoxically promotes tumor progression in established cells by providing nutrients (36, 55). It is noteworthy that in cancer cells, autophagy suppresses the immune system by affecting T cells, cytokines, and other immune system cells. In fact, autophagy helps the immune system by removing damage-associated molecular patterns (DAMPs) and pathogen-associated molecular patterns (PAMPs). Thus, the immune system is prevented from functioning properly in cancerous health conditions due to the increase in autophagy and removal of excessive DAMPs and PAMPs. In other words, the function of T cells, NK cells, cytokines, and other vital parts of the immune system is overshadowed by complicated processes of autophagy (56, 57).

In preclinical breast cancer models, autophagy has performed a strong role in the survival of quiescent disseminated cells (58). Compared to normal tissues, breast cancer cells hold a low level of Beclin1 proteins (59). Human *BECN1* monoallelic deletions are reported in up to 50% of breast cancers and 75% of ovarian cancers (60, 61). Numerous studies in the area of breast cancer survivors on the METABRIC (Molecular Taxonomy of Breast Cancer International Consortium) dataset demonstrated that a lower expression of *BECN1* is associated with a worse probability of survival (61).

The defective activity of any autophagic genes may affect carcinogenesis. It is detected that the mono-allelically deleted Beclin-1, as a haploid tumor suppressor, has an insufficient function in different cancers such as human hepatocellular carcinoma and breast, ovarian, and prostate cancers (62). Notably, many studies on ATG genes have demonstrated that ATG2B, ATG5, ATG9B, ATG12, and ATG16L1 are also oncologically linked to tumorigenesis. Furthermore, the somatic mutations in the ATG5 gene ruin the binding sites of ATG5 and Atg16L1, resulting in an impaired conjugation site for ATG12, and autophagy failure (63).

Evidence shows that in some situations, inhibition of autophagy and, in some cases, induction of autophagy has been beneficial for the treatment of cancers, depending on the tumor type or the stage of disease (64). Autophagy-dependent genes and proteins are valuable markers in tissue samples of patients for better diagnostics (65).

### 3.3 Autophagy and drug resistance

Several studies indicate that autophagy has a critical role in the survival of tumors and drug resistance. Obviously, autophagy is induced by chemotherapeutic drugs and then prepares the tumor cells for the nutrients by degradation of unwanted proteins or organelles. Accordingly, autophagy prevents DNA damage and enhances drug resistance in tumors (66). Although the exact mechanism of drug resistance with autophagy induction is not fully understood, some reports have suggested the role of autophagy in the induction of DNA damage response and the increase of drug efflux pumps in tumor cells (67). In this regard, autophagy may be involved in apoptosis inhibition by the inactivation of pro-apoptotic factors or the activation of anti-apoptotic agents. In this case, autophagy induction is used to overcome drug resistance. Prolonged autophagy promotes autophagic cell death (type II cell death), which is independent of apoptosis (type I cell death) or necrosis (type III cell death). In tumors, when the cells resist apoptosis, induction of autophagic cell death is an approach to trigger drug resistance. There are some drugs (like bortezomib, rapamycin, and butyrate) that induce autophagosome formation (68). However, considering the dual role of autophagy in cancer, another approach to target tumor drug resistance is autophagy inhibition. The use of gene silencing of autophagy-related genes or chemical inhibitors such as bafilomycin A1, 3-methyladenine (3-MA), and chloroquine (CQ) is commonly used to inhibit autophagosome formation (69).

### 3.4 Autophagy modulation

Enhanced autophagy in tumors, as a survival mechanism and a cause of MDR, leads to the use of autophagy inhibitors to suppress tumor cell proliferation and induce cell death. The application of autophagy inhibitors in combination with antitumor drugs sensitizes the cancerous cells to chemotherapy (70). For example, treatment of HER-positive breast cancer cells with depletion of Atg5, Atg7, or beclin1 resulted in enhanced effectiveness of tamoxifen or the combination therapy of 3-MA, as an autophagy inhibitor, and trastuzumab, as a chemotherapeutic drug, increasing the effectiveness of chemotherapy in HER2-positive breast cancer cells as compared to monotherapy (71).

However, the use of anticancer drugs induces autophagy as a survival mechanism. However, therapies involving both excessive autophagy induction upon cytotoxic drug treatment or using autophagy inducers may also lead to autophagic cell death (72, 73). Accordingly, there are some autophagy inducers that have been used as anticancer treatments. For example, proteasome inhibitors (PIs) have also been shown to stimulate autophagy. Bortezomib, a PI used in the treatment of multiple myeloma and mantle cell lymphoma, has been shown to increase the early formation of autophagosomes and LC3-II, demonstrating its inducing effects on autophagy (74). A well-known class of autophagy inductors includes analogs of the mTOR inhibitor rapamycin, such as temsirolimus and everolimus. These compounds, which can be used alone or in combination with chemotherapy drugs, show an anti-proliferative effect in mantle cell lymphoma and acute lymphoblastic leukemia by overstimulating autophagy, which might cause tumor cell death. A large number of ongoing trials demonstrate that autophagy modulation in combinatory treatments could successfully overcome the resistance to existing anticancer drugs. For instance, RUBCN (Rubicon autophagy regulator), as a negative regulator of autophagosome biogenesis, leads to the inhibition of basal differentiation and attenuates metastatic growth *in vivo* (5, 73). There are many FDA-approved and unapproved autophagy modulatory drugs that are introduced for cancer treatment. For example, CQ and hydroxychloroquine (HCQ) are determined as autophagy inhibitors, rapamycin and metformin are used as autophagy inducers in various cancers, and CQ and metformin are mainly used in breast cancer. The mechanism of CQ is suppressing the fusion of autophagosome and lysosome, while metformin activates AMPK (74) through ROS production in the mitochondria and inhibition of the mitochondrial respiratory chain complex 1 (75).

### 3.5 Breast cancer treatment by autophagy modulation

Since autophagy has a significant role in the survival of cancer cells, its modulation is considered a hopeful strategy for cancer treatment. Previous studies have shown that autophagy is the main reason for resistance to chemotherapy in breast cancer and has resulted in decreased sensitivity to chemotherapy with doxorubicin (DOX). Experiments indicated that the use of autophagy inhibitors can reverse doxorubicin resistance and enhance its efficacy in triple-negative breast cancer MDA-MB-231 cells (76). In addition, the cotreatment of DOX and 3-MA resulted in a necroptotic form of cell death in breast cancer (76). Using CQ is another approach to return DOX sensitivity in breast cancer MCF-7 cells (77).

Overexpression and accumulation of LAMP2A were observed in breast cancer tissue samples, and its inhibition conferred sensitivity to DOX in MCF-7 and T47D breast cancer cells (78). A novel adipokine, resistin, is highly stimulated in patients with breast cancer and facilitates cell proliferation, metastasis, and breast cancer cell migration. Resistin can activate AMPK/mTOR/ULK1 and JNK signaling pathways. Interestingly, the blockage of the two mentioned pathways reduces the ratio of LC3-II/LC3-I, which grants increasing apoptosis in breast cancer cells induced by DOX (79). In addition, in DOX-resistant breast cancer cells, psammaplin A (a natural product isolated from marine sponges with anticancer effects) can stimulate overexpression of damage-regulated autophagy modulator (DRAM) that is induced by p53 protein (80). In estrogen receptor-positive breast cancer, attenuated autophagy sensitized resistant tumors to tamoxifen-induced cell death (81). The summary of some autophagy inhibitors and inducers that are used in breast cancer is listed in **Tables 1**, **2** respectively.

**Table 1:** The compounds with autophagy inhibition characteristics.

Compound	Mechanism of action	References
SB02024	The compound is a VPS34 inhibitor; it blocks autophagy and increases the sensitivity of breast cancer to sunitinib and erlotinib drugs	(6)
3-Methyladenine (3-MA) or bafilomycin A1	The combination of 3-MA and gefitinib (Ge) enhance the effect of treatment in triple-negative breast cancer *in vitro* and *in vivo*. An increased level of BAX/Bcl-2, cytochrome *C* and CASP3 has been observed	(82)
Tioconazole	An antifungal drug that inhibits ATG4B and autophagy; it induces sensitivity to chemotherapy	(83)
3-MA	3-MA is an autophagy inhibitor. However, its combination with anticancer drugs is autophagy-independent	(84)
(2*S*)-8-[(3*R*)-3-Methylmorpholin-4-yl]-1-(3-methyl-2-oxobutyl)-2-(trifluoromethyl)-3,4-dihydro-2*H*-pyrimido[1,2-*a*]pyrimidin-6-one	The compound is a VPS34 inhibitor and suppresses autophagy pathway	(85)
Mefloquine (MQ)	The drug inhibits autophagy through lysosomal function disruption; induces endoplasmic reticulum stress and apoptosis in both hormone receptor-positive or receptor-negative breast cancer cell lines	(86)
IITZ-01 and IITZ-02	They inhibit maturation of lysosomal enzymes and increase accumulation of autophagosomes, which leads to autophagy inhibition	(87)
GDC-0941	The compound is a PI3K inhibitor. The combination of GDC-0941 and an autophagy inhibitor enhances the sensitivity of estrogen receptor (ER)-positive breast cancer to treatment	(88)

**Table 2:** The compounds with autophagy induction characteristics.

Compound	Mechanism of action	References
LYN-1604	The compound activates ULK1 and autophagy through apoptosis induction in triple-negative breast cancer.	(89)
Metformin	Metformin inhibits the mTOR effector, p70S6K1, and induces AMPK activity.	(90)
Tat-BECLIN1	The compound blocks the HER2/Beclin1 binding and enhances autophagy in HER2-positive breast cancer.	(91)
Paratocarpin E	The compound induces autophagy by increasing the ratio of LC3-II/LC3-I and Beclin-1 levels; it induces apoptosis *via* alteration of Bax and Bcl-2 expression; it activates caspase-8, caspase-9, and PARP cleavage.	(92)
Sarsaparilla (Smilax Glabra Rhizome) extract	The compound induces autophagy *via* ERK1/2 pathway and also inhibits cancer growth through apoptosis induction in breast cancer cell line.	(93)
Isoliquiritigenin (ISL)	The compound induces autophagy through miR-25 overexpression and ULK1 activation.	(94)
Gelomulide K	The compound increases the paclitaxel effects through reactive oxygen species (ROS) production, autophagy induction, and caspase-independent cell death.	(95)
Juglanin	The compound induces autophagy through the ROS/JNK signaling pathway and subsequent apoptosis activation.	(96)
CYT-Rx20	The compound induces autophagy through activation of Beclin-1, ATG5, and LC-3 proteins and also induces apoptosis.	(97)
Resveratrol	The compound induces autophagy through inhibition of the breast cancer stem cell and Wnt/β-catenin signaling pathway.	(98)

## 4 Autophagy modulations by nanoparticles and nanocarriers

Most of the autophagy modulators that are currently available have low specificity, as they do not preferentially target a single cell type. In addition, multiple autophagy mediators contribute to several cellular processes and are involved in autophagy-independent functions. For instance, rapamycin, as a well-known autophagy inducer, has an impact on the inhibition of T-cell proliferation, which results in strong immunosuppression (99). These challenges lead to limitations in the use of autophagy regulators in cancer treatment. In this regard, NPs have multiple advantages to overcome these issues. They can improve the efficacy of drugs by their high power of permeability and reduce their toxicity due to nanosized properties. They lead to more effective targeting of tissue, cells, or organelles and enhance the pharmaceutical properties of drugs such as stability, solubility, plasma half-life, and tumor accumulation (7). NPs can be used alone or in combination with drugs. However, NC is a nanomaterial that is used as a transport mediator for other substances, such as a drug. Generally, NCs are categorized as micelles, polymers, carbon-based materials, liposomes, and other materials and are used in drug delivery, especially in chemotherapy (100).

The connection between NPs and their impact on autophagy has shown that they can impact autophagy through both its induction and inhibition. The induction of autophagy is mainly mediated through oxidative stress activation. Under external stress, the phagocytosis of NPs or NCs is enhanced, and mitochondrial respiration is also increased. Hence, the accumulation of a large number of incompletely reduced oxygen atoms results in the generation of a large number of ROS, which leads to programmed cell death (101). Autophagy induction by NPs may be a defensive mechanism against external signals. The use of CQ and NPs enhances the effectiveness of chemotherapy by the accumulation of NPs in the tumor and decreases the immunological clearance of NPs. The NPs can directly reach the tumor cells; therefore, they can reduce the non-specific functions of autophagy regulators, enhance the accumulation of drugs at tumor sites, and consequently enhance the antitumor efficacy (89).

Although NPs have several advantages in biomedical applications, they have shown some cytotoxic effects on cancerous cells. For example, inflammation, ROS production, apoptosis, or necrosis occurs following the toxicity of NPs, limiting their application. To overcome these barriers, some modifications are necessary in order to increase the efficacy of NPs. For example, changing the dose of treatment or modifying their shape or formulation could reduce the toxicity level. The dose of NPs used in treatment is important in their aggregation and distribution. Moreover, the direct administration of NPs on the skin is less toxic than intravenous injection. Consequently, conducting an accurate protocol for using NPs in biomedicine is critical (90).

**Figure 3:**
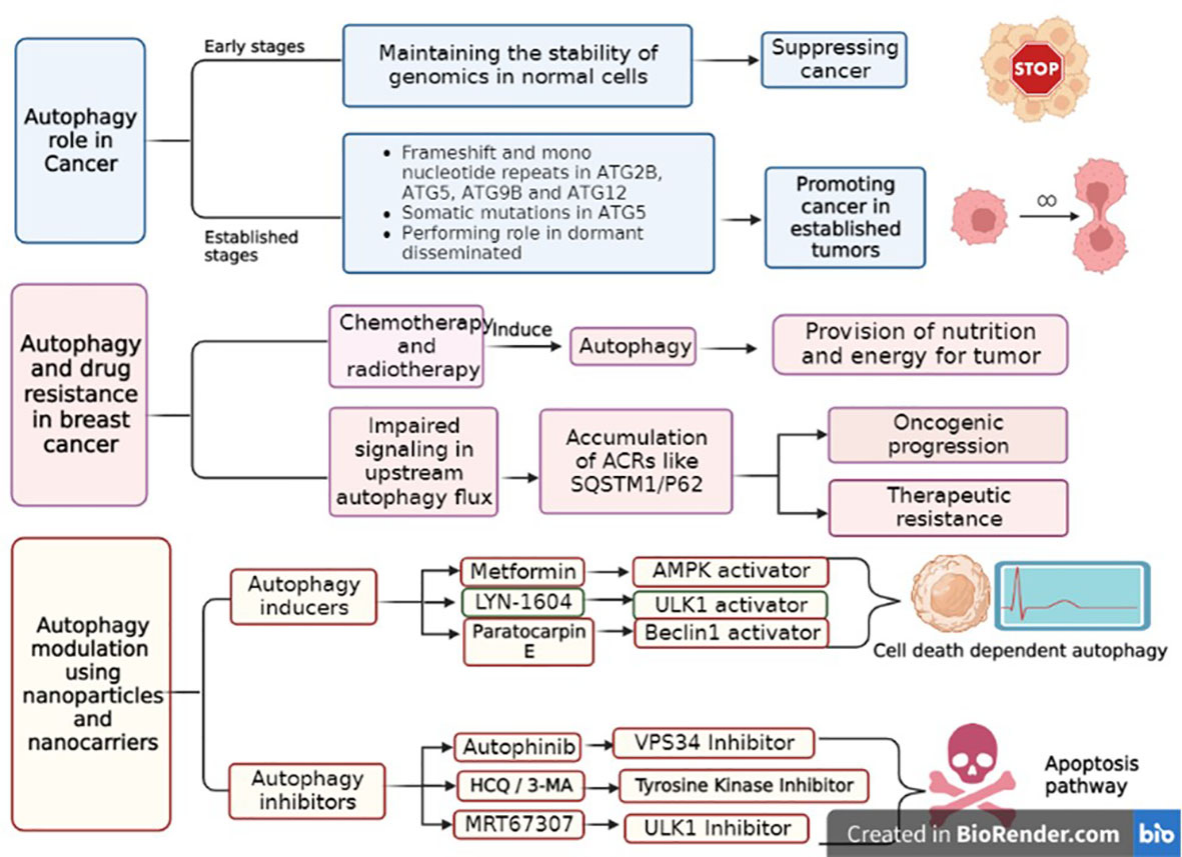
The role of autophagy in breast cancer, its drug resistance and the approaches of autophagy pathway modulation. Autophagy plays a dual role in cancers, according to the stage of tumors. In general, it acts as a tumor suppressor in early stages, while in established tumors, it promotes tumor progression. During chemotherapy or radiotherapy, autophagy is activated to provide nutrients for tumors and induce drug resistance. Autophagy inducers lead to autophagy activation, which finally promotes cell death in an autophagy-dependent manner. In contrast, autophagy inhibitors suppress the nutrient pathway for tumors, ultimately leading to apoptotic cell death.

The different effects of NPs on autophagy, induction, or inhibition, in different situations, lead to antitumor activity or an increase in cell death (89). In practice, the choice between inhibition and promotion of autophagy is controversial, as it may depend on the role of autophagy in tumor development. As long as autophagy exerts a positive effect on the treatment of certain cancers, strategies that promote autophagy remain desirable. However, when autophagy adversely affects cancer treatment, inhibition of autophagy is the appropriate strategy. Depending on the type of cancer, therapy should involve an appropriate treatment in combination with autophagy modulation (72). **Figure 3** illustrates the role of autophagy in cancer, drug resistance, and the modulation of autophagy in breast cancer.

### 4.1 Nanocarriers

NCs, as a specific type of drug delivery system, can target tumor cells through passive and active targeting methods and accumulate more in cancerous tissue, compared to normal tissues. Passive targeting means the direct permeation of drugs into tumor tissue, which depends on the action of the enhanced retention system or enhanced permeation system (EPS) effect. As a tumor develops in size and shape, an increased demand for oxygen and nutrients leads to the requirement for new blood vessels. These vessels are often not properly developed and are, therefore, permeable to some particles of certain sizes, usually below 700 nm (90). To function properly, NCs should be less than 100 nm in diameter and have a naturally hydrophilic surface, indicatively to avoid macrophage clearance and adhesion to plasma proteins. Polyethylene glycol, polysaccharides, poloxamines, poloxamers, and amphiphilic copolymers are some NCs with passive targeting ability (102). In active targeting, NCs are coated with specific ligands that can recognize their targets on the surface of cancer cells (91). NCs can be transferred directly to the cancer cells mainly through the following mechanisms. First, they can be delivered directly to the cancer cells through carbohydrate targeting, as the tumor cells have more carbohydrates in their membranes than normal cells. The second approach to the delivery of NCs is through receptor targeting. Some of the NCs are equipped with ligands of specific tumor cell receptors to recognize the tumor cells. In this method, after connecting the NCs to the tumor receptors, the drug is dissociated from the carrier and transferred to the target sites (92).

There are structurally different types of NCs explored for the effective treatment of breast cancer, including polymeric micelles, dendrimers, nanoliposomes, carbon nanotubes, solid lipid NPs (SLNs), nanostructured lipid carriers (NLCs), nanoemulsions (NEs), gold-based NPs (AuNPs), protein nano cargoes, and aptamers (93). In **Table 3**, we discussed the structure and properties of each nano-drug delivery system in the breast cancer experimental models (94).

**Table 3:** The application of different types of nanocarriers (NCs) with their structures and characteristics in breast cancer experimental models.

Systems	Structure	Characteristics	References
Polymeric micelles	Amphiphilic in nature; hydrophobic core and hydrophilic shell	Biocompatible and biodegradable; self-assembly and functional modification capability; active and passive targeting	(93)
Dendrimers	Synthetic, uniform structures, composed of core, branches, and surface regions	Equity in size, shape, and the length of branches; enhanced surface area, loading capacity, and targeting ability; improve pharmacokinetics and biodistribution of drugs	(95)
Liposomes	Lipid bilayer membrane forming self-assembled closed colloidal structures with an aqueous core	Biocompatible and biodegradable; providing improved pharmacokinetics altered biodistribution of the drug; sustained and slow release of the drug; can deliver hydrophilic, hydrophobic, and amphiphilic drugs	(96)
Carbon nanotubes	Cylindrical nanoshape structures made of allotropes of carbon a) single-walled carbon nanotubes (SWCNTs), with one layer of graphene sheet and b) multiwalled carbon nanotubes (MWCNTs), with multiple layers of SWCNTs coaxially arranged	Multiple functions; high entrapment efficacy; monodispersity, feasibility of synthesis and sterilization; chemical modification; water-soluble, biocompatible; ability to incorporate any functional groups; active or passive targeting; showing prolonged distribution and localized effects	(97)
Solid lipid nanoparticles (NPs) (SLNs)	A surfactant layer on the surface with a lipid matrix consistent with solid lipid (s)	Biocompatible and biodegradable; non-toxic; high stability and feasibility of scale-up; high drug loading; reduced toxicity, enhanced bioavailability of poorly water-soluble and bioactive agents; targeting; capable of loading hydrophobic and hydrophilic drugs	(98)
Nanostructured lipid carriers (NLCs)	Second generation of SLNs; composed of a surfactant outer layer and solid lipid matrix along with a liquid lipid	Higher drug encapsulation and loading compared to SLNs	(103)
Nanoemulsions (NEs)	Droplets of water and oil dispersed biphasically and stabilized by an amphiphilic surfactant	Higher solubility than micellar dispersions; long-term physical stability; passive targeting with enhanced permeability and retention (EPR) effect; they can carry very hydrophobic drugs, improving their bioavailability	(104)
Gold-based NCs (AuNCs)	Different structures, including nanocubes, nanospheres, nanorods, nanoshells, nanobranches, nanocages, and nanowires	The most stable NPs; capable of active and passive targeting; can be PEGylated easily; scattering and light absorption characteristics when exposed to near-infrared wavelength (NIR) and heat production, which can ablate tumor cells	(105)
Protein nanocages	Shell-like containers, with intrinsic homogeneous chambers circumscribed by protein walls; have three distinct surfaces: exterior and interior surfaces, the interfaces between subunits	Smaller particles can deliver targeted therapy; monodisperse, biocompatible, water-soluble, biodegradable; selective for cancer cells; extremely homogenous size distribution; can be efficiently produced by genetic engineering	(106)
Aptamers	Single-strand oligonucleotides	Feasibility of synthesis and modification; showing low immunogenicity and efficient delivery to different types of cells; modification with siRNAs, miRNAs, and anti-miRNAs can serve for gene delivery	(107)

### 4.2 Nanoparticles

NPs are commonly described as particulate subjects at least smaller than 100 nm in one dimension (108). NPs are structurally different from each other and include liposomal NPs composed of phospholipids (109); polymeric NPs synthesized from natural products like gelatin, albumin, or artificial polymers (e.g., polylactides); poly alkyl cyanoacrylates; inorganic NPs involving silica NPs; quantum dots; and metal NPs. In addition, other properties such as size, shape, surface charge, and inflexibility are important when choosing NPs as treatment tools. These properties impact the NPs’ cellular uptake through reticuloendothelial systems, targeting the right cells and tissue distribution (110). In the following, the physiochemical properties of NPs are explained in detail (109).

#### 4.2.1 Size of NPs

The size of NPs is a significant factor in drug delivery and activation of the immune system and also modulates the pharmacokinetic actions of therapeutic agents, such as cell uptake, biodistribution, and body fluid half-life (111, 112). For example, gold-based NPs with a size of 10 nm have a longer blood circulation time and a reduced accumulation in the liver and spleen, compared to a size of 20 nm. Large NPs with a diameter between 100 and 200 nm are needed to deliver enough medication to disease sites (111). NPs from 30 to 50 nm exhibit efficient cellular absorption due to increased specific surface area and membrane encapsulation process. Phagocytic activity is considered to be the main pathway by which larger NPs from 250 to 3000 nm in size are internalized into the cells. Glomerular capillary walls have physiological pores between 4.5 and 5 nm; therefore, NPs smaller than 6 nm are effectively filtered, while those larger than 8 nm cannot be eliminated by glomerular filtration (113).

The 100–200-nm NPs, including microparticles, are useful for drug loading, considering their high capacity. However, these large particles undergo active opsonization, readily trigger immune responses, and accumulate rapidly in the liver and spleen, thereby exhibiting poor systemic circulation (114). Small NPs lower than 5 nm should be considered not only because of their low carrying capacity but also because of their fast rate of renal clearance. However, many studies have found that small NPs are advantageous in several respects compared to their larger counterparts, including minimal immunogenicity, long systemic circulation, and easy entry and aggregation in the tumor, as well as independence for caveolin-dependent cell pathways uptake (114). Because of their excellent absorption efficiency and significant tumor accumulation, NPs with a size of 30–200 nm have been widely used in numerous investigations (113).

#### 4.2.2 Shape of NPs

NPs can be used in a variety of nanomaterials, including liposomes, micelles, dendrimers, and metal NPs. The ability of NPs to interact with cell membranes depends on their shape. For instance, non-spherical NPs might yield multivalent interactions with cell surface receptors, whereas spherical ones only interact with a small number of binding site receptors (115).

#### 4.2.3 Surface charge

The plasma membranes of the cells show a moderate negative voltage difference, varying from −90 to −20 mV from cell to cell. The pharmacokinetics and blood circulation time of NPs are influenced by the electrostatic interactions between NPs and the proteins (115). For instance, cationic polymeric NPs encapsulating tetrandrine had superior anticancer activity in A549 cells and a higher cellular absorption efficiency than anionic NPs (116).

#### 4.2.4 Elasticity

It has been thoroughly investigated whether the elasticity of NPs plays a significant physicochemical role in determining their pharmacokinetics and biodistribution. According to certain studies, the flexibility of NPs affects how they interact with tissues and immune cells (117). Additionally, the rigidity or softness of NPs is an important factor in their distribution. The uptake of the stiffer NPs is shown to disrupt the architecture of the cell, while soft NPs can enter the cells through micropinocytosis easily (118). The absorption process is thought to have changed from fusion, which has a low energy dependence, to endocytosis, which has a high energy dependence due to the differential uptake of soft and stiff NPs by cells (117). Soft NPs have a longer half-life in the bloodstream due to the lower absorption by macrophage cells, which is also reliant on their elasticity (117). Commonly, a significant factor that affects the pharmacokinetic behavior of NP therapies is their elastic modulus. It has been demonstrated that elastic modulus optimization affects the interactions of NPs with different cells and their circulating half-life, tumor targeting, and aggregation effectiveness (119).

### 4.3 NPs and autophagy modulation

**Figure 4:**
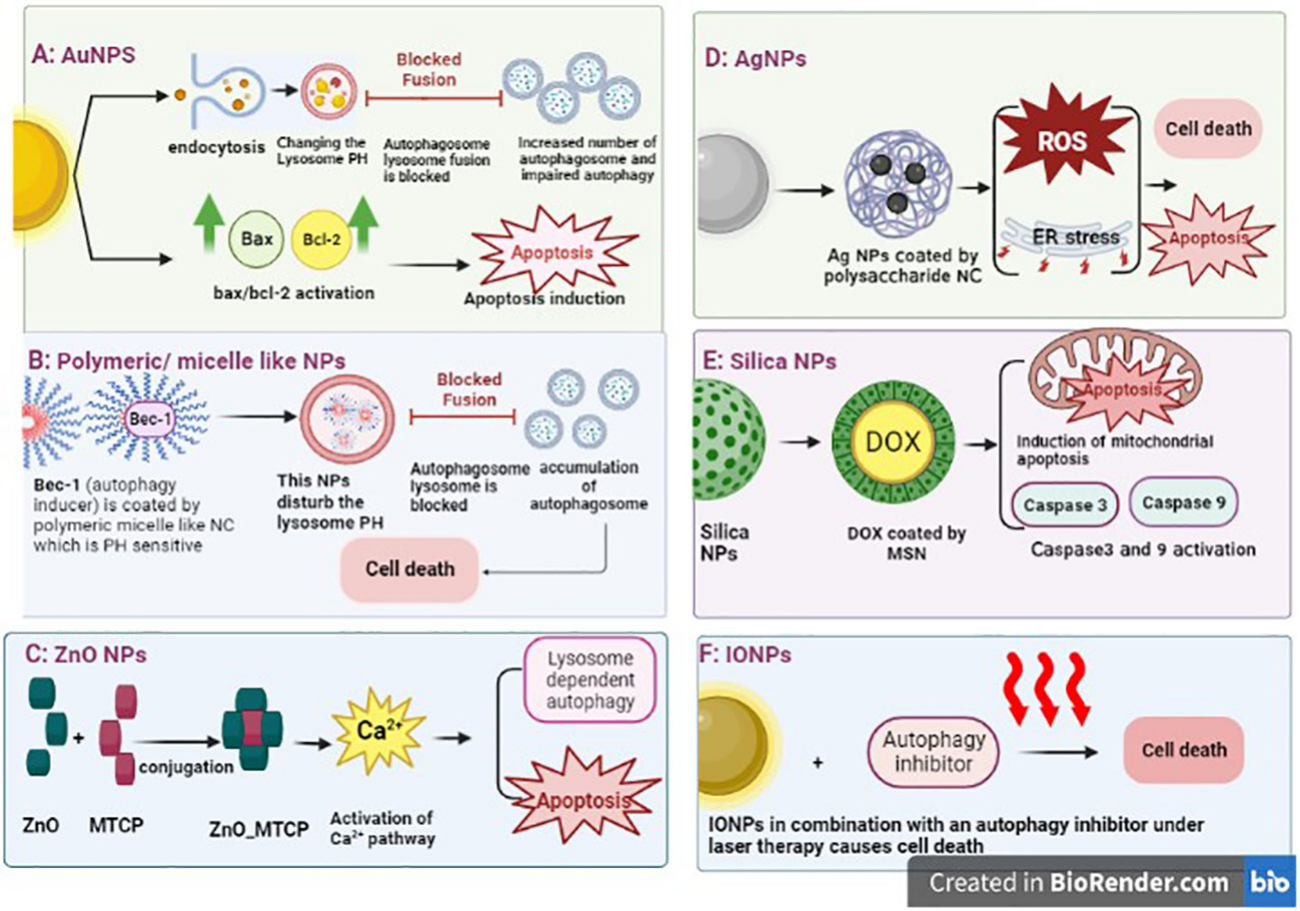
Different nanoparticle-mediated autophagy. **(A)** AuNP delivery system enters the cells, accumulates in the lysosome, and changes its pH. The impairment of lysosome function leads to accumulation of autophagosomes in cells without fusion with lysosomes. Then, apoptosis is induced. Another study has shown the effect of NPs on the Bax/Bcl2 activation that leads to apoptosis. **(B)** Polymeric/micelle-like NPs can encapsulate the autophagy inducer that disturbs the pH of lysosome leading to cell death. **(C)** ZnO can conjugate with drugs and impact calcium pathway, which ultimately leads to autophagy. **(D)** AgNPs induce autophagy through activation of Atg5 expression, which can result in cell death through autophagy or apoptosis induction. **(E)** Silica NPs can encapsulate chemotherapeutic drugs, such as doxorubicin, and induce intrinsic apoptosis through mitochondria. **(F)** The effect of photothermal therapy of IONPs in breast cancer cells has been observed through autophagy induction, independently of apoptosis. The use of autophagy inhibitors and IONPs under laser therapy causes cell death. AuNP, gold-based nanoparticles; AgNPs, silver-based nanoparticles; IONPs, iron oxide nanoparticles.

Considering the aforementioned data, autophagy modulation may be a promising treatment, alone or in a combination with other therapeutic methods to combat resistance and ensure a successful treatment of breast cancer. However, most current autophagy regulators suffer from low specificity, poor targeting, and bioavailability, as they are insufficiently solubilized in aqueous media and cannot specifically target tumor tissues (120). Leveraging nano-drug delivery systems reduces toxicity and increases drug efficacy through more appropriate targeting (121). The NPs being studied for drug delivery can be divided into different categories considering the type of therapy in which they are implemented. Three major therapeutic NPs are chemotherapeutic, phototherapeutic (including photodynamic therapy, and photothermal therapy), and immuno-therapeutic NPs, along with other categories such as sonodynamic therapeutic NPs. Each of the categories induces cytotoxicity *via* different intracellular mechanisms such as inhibition or induction of autophagy (72, 121, 122). NPs are divided into three categories: metal, organic polymer, and inorganic non-metallic NPs (115). The NP-based drug delivery into the cell is schematically illustrated in **Figure 4** and is discussed in detail in the following section.

#### 4.3.1 Silver-based NPs

Silver-based NPs (AgNPs) have been shown to induce autophagy in breast cancer cells, ultimately leading to apoptosis. One experiment that used the AgNPs embedded in specific polysaccharides showed good entrance to the cells, generation of ROS, and induction of endoplasmic reticulum stress, leading to cell death through autophagy or apoptosis (123). The effect of AgNPs on cytotoxicity in breast cancer cells also confirmed that regardless of the shape and structure of the AgNPs, they are cytotoxic for triple-negative breast cancer (TNBC) *in vitro* and xenograft model but have no effect on non-malignant cells. Hence, the application and development of AgNPs in breast cancer treatment should be safe (124).

#### 4.3.2 Gold-based NPs

The effect of gold-based NP (AuNP) cytotoxicity on cells has a different intracellular mechanism. AuNPs enter the cells by endocytosis, accumulate in the lysosome, and change the pH value. Impaired lysosome function has implications for the autophagy pathway. Indeed, AuNPs induce the accumulation of autophagy in cells; however, the formation of autophagy flux is blocked due to an impairment of lysosome degradation (125). Another research showed that induction of apoptosis in AuNP-treated breast cancer cells occurred through p53 and bax/bcl-2 activation (126).

#### 4.3.3 Zinc oxide NPs

The lethal toxicity of zinc oxide NPs (ZnO NPs) on cells could be modified by NP stabilization. An experiment with the conjugation of ZnNPs with porphyrin (MTCP) yielded a cytotoxic effect of NPs in breast cancer cell lines through the calcium signaling pathway, which regulates lysosomal-dependent autophagy and apoptotic cell death (127).

#### 4.3.4 Iron oxide NPs

The effect of photothermal therapy of iron oxide NPs (IONPs) in breast cancer cells was observed by inducing autophagy but not leading to apoptosis. The use of autophagy inhibitors and IONPs under laser therapy causes cell death in both MCF-7 and xenograft breast cancer models (128).

#### 4.3.5 Copper oxide NPs

The cytotoxicity of using copper oxide NPs (CuONPs) in MCF3 breast cancer cells was observed *via* oxidative stress and autophagy induction. In these cells, autophagy is activated in order to survive the tumor cells. Therefore, the cotreatment of autophagy inhibitors and CuONPs could be a potent treatment against breast cancer cells (129).

#### 4.3.6 Silica-based NPs

The use of nano-drug delivery in cancer cells has been demonstrated to improve biodistribution and increase the sensitivity of the drugs. For instance, the use of DOX-loaded mesoporous silica nanoparticle (MSN) in breast cancer treatment has shown an increased level of apoptosis, in comparison with DOX treatment alone in both *in vitro* and *in vivo* experiments. The intracellular mechanisms also confirmed the induction of the pro-death autophagy signaling pathway through inhibition of the AKT-mTOR-p70S6K signaling pathway (130). Another study has shown the cytotoxic effects of silica-based NPs (SiNPs) in breast cancer cell lines through the mitochondrial apoptotic pathway. The NPs with 5–15 nm could induce caspase-9 and caspase-3 activities (131).

#### 4.3.7 Polymeric NPs

Synthetic pH-sensitive polymeric NPs have been used to increase chemical stability and specific biodistribution. For instance, the Bec-1 peptide, as an autophagy inducer, was engineered into the pH-sensitive polymers and then self-assembled with polyethylene glycol to form micellar-like NPs. This structure of NPs alters the acidic status of lysosomes and prevents autophagosome–lysosome fusion. Accumulation of autophagosomes leads to cell death, not tumor survival (132). In **Table 4**, we have summarized several studies looking at the implementation of an autophagy modulator with a nano-drug delivery system, some of which were also combined with chemotherapeutic agents. In addition, it is shown that NCs are able to modulate autophagy, simultaneously targeting cancer cells and delivering therapeutic agents.

**Table 4:** The compounds with autophagy modulation properties in breast cancer.

Autophagy modulator	Combined chemotherapeutic agent	Nano-delivery drug system	Details	References
Chloroquine (CQ) phosphate (inhibition)	Doxorubicin (DOX); docetaxel (DTXL)	Block copolymer PEG5K-*b-*PLA8K (PEG-PLA)	1. DOX or DTXL induce autophagy and lead to resistance in breast cancer stem cells (CSCs). Inhibition of autophagy promotes efficiency of chemotherapy 2. Cotreatment of CQ with wortmannin and 3-methyladenine (3-MA) showed enhanced effect 3. A nanoparticle delivery system increases drug accumulation in tumors and CSCs *in vitro* and *in vivo*	(133)
CQ		Gold-based NPs (AuNPs)	Drug release at acidic PH environment; autophagic death of MCF-7 cells	(134)
Hydroxychloroquine (HCQ) (inhibition)		CCM-HMTNPs	HMTNPs enable active targeting by being conjugated with cancer cell membrane (CCM) and are sensitive to ultrasound (sonodynamic therapy (SDT)); generate ROS when activated; HCQ blocks autophagy flux and eliminates resistance to SDT	(135)
SiRNAs (inhibition)	Paclitaxel	Polymeric micelles or NPs	SiRNAs target autophagic proteins and mRNAs	(136)
Bec1 (induction)		pH-sensitive poly(β-amino ester)s-PEG (micelle)	By covalently linking the nanocomposites with Beclin1, higher drug uptake efficiency and enhanced autophagy induction are achieved; P-Bec1 (polymer beclin1) displays enhanced cytotoxicity to breast cancer cells through induction of autophagy	(132)
Primaquine (PQ) (inhibition)		Nanocapsule HCG	HCP@PQ/ICG is a combination of PQ and ICG (a photothermal agent), which combats cytoprotective autophagy induced by phototherapy	(137)
Berberine (BB)		Au-Col	Au-Col consists of gold NPs fabricated with biocompatible collagen; Au-Col-BB shows enhanced cellular uptake and significant inhibition of cell migration, expression of apoptotic cascade proteins, and a remarkable decrease in tumor weight	(138)
Salinomycin (SAL) (induction)	DOX	Liposomes	SAL is an antibiotic with potency of autophagy and subsequent apoptosis induction; this combination is able to eliminate both CSCs and breast cancer, thus preventing recurrence of tumor and showing complete response	(139, 140)
Dihydroartemisinin (DHA) (induction)	Epirubicin	PEGylated liposomes	Increased anticancer effects in treatment of heterogeneous breast cancer	(141)
CQ	DOX	Liposomes	Higher efficacy with respect to liposomal DOX or free DOX	(142)
Thymoquinone (TQ) (induction or inhibition, according to cell type)	DTXL	Chitosan-grafted lipid nanocapsules; PEGylated liposomes	Implication in drug-resistant breast cancers, triple-negative breast cancers (TNBCs), and metastatic breast cancers; increased circulation time and enhanced cytotoxicity	(143)
CQ		PAMAM-DEACM	PAMAM is an NC with anticancer capability; it was modified with a photocleavable curamin (DEACM), loaded with CQ, and used to combat the cytoprotective autophagy induced by PAMAM	(144)
Ginsenosides (induction)		Polymeric NPs, liposomes, protein-based nanocarriers, etc.	Low bioavailability can be improved by NPs. It can be conjugated to liposomes to enhance stability and avoid adverse effects and can be used as a biological drug delivery system. View reference for detailed information	(145)
Rapamycin (Rap) (induction)	Epothilone B (EpoB)	Bioresorbable micelles functionalized with biotin (PLA-PEG-BIO)	Combination therapy with Rap with EpoB was very effective in reducing cell metabolic activity and survival. PLA–PEG-BIO enhanced the cytotoxic effects of these agents	(146)
TQ	DTXL	Borage oil-based nanoemulsion (B-NE)	Treatment with DTX, along with other therapeutic effects, promotes autophagic death in MCF-7. (DTX + TQ) B-NE can increase the autophagic cell death values significantly	(147)
Artemisinin (ART)	TF; paclitaxel; epirubicin; daunorubicin	Inorganic NPS; liposomes; micelles; polymer-based NPs; carbon-based NPs; niosomes	ART induces regulated cell death mechanisms, such as apoptosis, ferroptosis, autophagy, necroptosis, and pyroptosis. ART-related NC can resolve low solubility, low bioavailability, short plasma half-life, and chronic toxicity of ART. View reference for detailed information	(148)
Bcl-2 and PKC-ι siRNAs (inhibition)		Aptamer-coupled QD-lipid nanocarriers (aptamo-QLs)	Simultaneous reduction of Bcl-2 and PKC-ι expression can synergistically inhibit TNBC cell proliferation and metastasis, and promote autophagic cell death	(107)
CQ		Triphenylphosphonim-functionalized dendrimer	A mitochondrial **tropictri** phenyl phosphonium-functionalized dendrimer manifested substantial cytotoxicity both alone and after encapsulation of chloroquine	(149)
CQ		Frizzled7 antibody-coated nanoshells (FZD7-NS)	Inhibition of Wnt signaling pathway with Frizzled7 antibody in TNBC can lead to upregulation of autophagy and cause therapeutic resistance. A combinational therapy of CQ with FZD7-NS led to inhibition of TNBC cell migration and self-renewal more effectively	(150)

## 5 The advantages and disadvantages of using nanomaterials in breast cancer treatment

NPs are an ideal drug delivery vehicle due to their nanosized structure, good biodistribution, and cost-saving properties (151), Furthermore, it is widely established that NCs aid in boosting the solubility, bioavailability, and stability of a variety of pharmacological drugs (152). Additionally, by improving cancer cell targeting, NPs could reduce the harmful effect of active cancer drugs. Finally, a safe and efficient agent for combination therapy involving autophagy regulation is one of the nano-drug delivery system advantages (153). Despite their small size, NPs can be easily and specifically delivered to the cells and absorbed by the tissue of interest. NPs may be used in conjugation with target drugs specific to cancer cells without disrupting normal cells’ structure or function. They have specific qualities, including high encapsulation efficiency, biocompatibility, reduced toxicity, and ease of manufacture, making them useful for medications (153). Moreover, NP-based therapy is useful for personalized medicine and biomarker identification. NPs are easily engineered to be pH or temperature sensitive in different situations to transport and release the drug to their specific targets. Despite all these advantages of NPs, not all of them enter the clinical phase because there are some problems like biological and technological difficulties in manufacturing NPs (154). The biological issues, including the route of administration (e.g., oral, intravenous, or skin injected), biological barriers, the toxicity of NPs, and their degradation are essential factors in NP efficacy. To overcome these challenges, some modifications are necessary to improve the NP application. For example, the use of low concentrations, which may decrease the toxicity effects, or the production of NPs with more biocompatible materials, such as chitosan, are beneficial (155). Technological challenges including the scale-up synthesis of NPs and *in vitro* or *in vivo* experimental models are not enough to directly use NPs in the clinical study due to little supporting data. In this way, using computational models or organs-on-chip can be a good solution alongside the laboratory results (154).

## 6 The future of NPs in breast cancer

NPs have a high potential to be used in the future treatments of breast cancer. They overcome the challenges of current treatments and combat drug resistance. They help increase the sensitivity of drugs (65, 156). In addition to therapy, nanomedicine may assist patients in some other aspects, such as diagnostics in the early stages and tumor imaging. Nanotechnology offers potential solutions to the problems that have made breast cancer so challenging to treat. These problems include the diversity of cancer and the rapid evolutionary changes in patients, the multiple pathways driving disease progression simultaneously, the emergence of tolerance, creating therapeutic cocktails, distant breast cancer metastasis, and the severity of side effects of therapies and very poor biodistribution of the injected medications in the body (72, 157). However, nanomedicine has some important challenges and controversies that should be resolved. The major issue is the toxicity of nanomaterials. For instance, ROS production leads to DNA damage and protein denaturation. By standard protocols, deep toxicological studies prior to the application of NPs for human therapy are suggested.

Among many nanomaterials in biomedicine, only a few of them have reached clinical trials and obtained FDA approval. The process by which a new drug passes all the preclinical phases and clinical trials to obtain the license for use in humans is estimated to be approximately 10–15 years (158). The preclinical phases are usually cellular testing, animal studies, toxicology control, safety, efficacy, and dose ranges of the new drugs (159). The clinical trials involve three phases, named I (testing dose and toxicity), II (safety and testing in a larger group), and III (randomized multicenter testing), that should be passed before introducing the new drugs to the market (158). Nanomedicine needs phase IV of post-marketing to consider the limitation or benefits of the application. In this regard, many studies are under investigation to obtain a license in order to enter clinical trials (160). According to clinical trials (www.ClinicalTrials.gov), there are currently 39 studies available that use different shapes and structures of NPs in breast cancer treatment, confirming the importance of using NPs in cancer treatment. Here, we summarize the 10 top studies in **Table 5**.

**Table 5:** Clinical trial of nanoparticles (NP)-based treatment strategies in breast cancer (https://clinicaltrials.gov)

Number	Nanoparticle	Status	Conditions
1	Albumin-Bound (Nab) Paclitaxel/Cyclophosphamide	Completed, Phase II	Early stage of breast cancer
2	Nanoparticle-based Paclitaxel vs Solvent-based Paclitaxel as Part of Neoadjuvant Chemotherapy	Completed, phase III	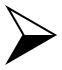 Tubular Breast Cancer Stage II 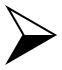 Mucinous Breast Cancer Stage II 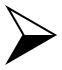 Breast Cancer Female NOS
3	Carbon NPs	Recruiting, phase not applicable	Lymph Nodes positive patients
4	Paclitaxel Albumin-Stabilized Nanoparticle	Completed, phase II	Metastatic breast cancer patients
5	Carboplatin, Paclitaxel, and Bevacizumab	Completed, phase II	Locally Recurrent or Metastatic Breast Cancer
6	Pertuzumab, Trastuzumab, and Paclitaxel Albumin-Stabilized Nanoparticle	Active but not recruiting, phase II	Patients With HER2-Positive Advanced Breast Cancer
7	Carboplatin and Paclitaxel Albumin-Stabilized Nanoparticle	Active but not recruiting, phase II	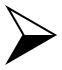 Inflammatory triple negative breast cancer 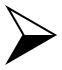 Stage IIA Breast Cancer 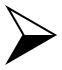 Stage IIIA Breast Cancer
8	Doxorubicin Hydrochloride, Cyclophosphamide, and Filgrastim Followed By Paclitaxel Albumin-Stabilized Nanoparticle Formulation With or Without Trastuzumab	Completed, phase II	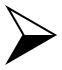 Estrogen Receptor-positive Breast Cancer 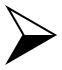 HER2-positive Breast Cancer 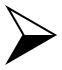 Stage IA Breast Cancer
9	Nanoparticle Albumin Bound Paclitaxel, Doxorubicin and Cyclophosphamide (NAC)	Terminated, phase I	Stages II-III Breast Cancer (NAC)
10	Paclitaxel Albumin-Stabilized Nanoparticle Formulation	Active not recruiting, phase II	Treating Patients of Different Ages With Metastatic Breast Cancer

## 7 Conclusion

NPs are an ideal drug delivery vehicle due to their nanosized structure, good biodistribution, and cost-saving properties. Additionally, by improving cancer cell targeting, NPs could reduce the harmful effect of active cancer drugs. It is challenging to modulate autophagy in breast cancer treatment utilizing NPs due to the different behaviors of autophagy in tumorigenicity. To deal with this problem, a large number of studies are recommended. The different effects of NPs on autophagy, induction, or inhibition, in different situations, lead to antitumor activity or an increase in cell death. In practice, the choice between inhibition and promotion of autophagy is controversial, as it may depend on the role of autophagy in tumor development. As long as autophagy exerts a positive effect on the treatment of certain cancers, strategies that promote autophagy remain desirable. However, when autophagy adversely affects cancer treatment, inhibition of autophagy is the appropriate strategy. Depending on the type of cancer, therapy should involve an appropriate treatment in combination with autophagy modulation. However, NPs have some disadvantages and cannot be used directly in clinical trials. To improve their lethal properties in cancerous cells, more modifications on NPs are needed, or cotreatment with targeted drugs is proposed. Despite some difficulties in the usage of NPs in medicine, their future application is promising and could be a new path for cancer treatment.

## Author contributions

AG has contributed as co-author for sections on breast cancer and autophagy mechanisms. She also designs the figures of the manuscript. DS has written the section on nanocarriers. AY has contributed and written the nanoparticles sections. FB has written the autophagy section and prepared the abbreviation section. MR has been invited by the editorial board of the Frontiers in Oncology, proposed the topic, and is a corresponding author of the manuscript. All authors contributed to the article and approved the submitted version.

## Glossary

**Table:**

3-MA	3-methyladenine
AgNPs	silver-based NPs
AMBRA-1	activating molecule in Beclin1-regulated autophagy
AMPK	AMP-activated protein kinase
ATG	autophagy-related genes
AuNPs	gold-based NPs
BCRP	breast cancer resistance protein
BCSCs	breast cancer stem cells
BECN1	Beclin1
CA15-3	cancer antigen 15-3
CEA	carcinoembryonic antigen
CMA	chaperon-mediated autophagy
CQ	chloroquine
DAMPs	damage-associated molecular patterns
DOX	doxorubicin
DRAM	damage-regulated autophagy modulator
DTXL	docetaxel
ER	estrogen receptor
ER-α	estrogen receptor α
ERK	extracellular signal-regulated kinase
FIP200	focal adhesion kinase interacting protein 200 kDa
GABARAPs	gamma aminobutyric acid receptor-associated proteins
GEMMs	genetically engineered mouse models
GSK3	glycogen synthase kinase 3
GTP	guanosine triphosphate
HCC	human hepatocellular carcinoma
HCQ	hydroxychloroquine
HER2	human epidermal growth factor receptor 2
HMGB1	high mobility group protein B1
IP3	inositol-3 triphosphate
JNK	c-Jun N-terminal kinase
Lamp2	lysosomal membrane-associated protein 2
LC3	microtubule-associated protein 1A/1B-light chain 3
MCF-7	Michigan Cancer Foundation-7 cell line
MDA-MB-231	highly aggressive, invasive and poorly differentiated triple-negative breast cancer (TNBC) cell line
MDR	multidrug resistance
MEK	mitogen-activated extracellular signal-regulated kinase
MQ	mefloquine
mTORC1	mechanistic target of rapamycin complex 1
NCs	nanocarriers
NLCs	nanostructured lipid carriers
NEs	nanoemulsions
NP	nanoparticles
PAI-1	plasminogen activator inhibitor
PAMPs	pathogen-associated molecular patterns
PARP	poly adenosine diphosphate-ribose polymerase
PAS	phagophore assembly site
PD-L1	programmed death-ligand 1
PE	phosphatidylethanolamine
PEG	polyethylene glycol
Pgp	P-glycoprotein
PI3K III(PI3KC3)	mammalian type III phosphatidylinositol-3 kinase
PI3P	phosphatidylinositol-3-phosphate
PKD	protein kinase D
PR	progesterone receptor
ROS	reactive oxygen species
SAL	salinomycin
SERCA	sarco-endoplasmic reticulum Ca^2+^-ATPase
shRNA	short hairpin RNA
SiNPs	silica nanoparticles
SLNs	solid lipid NPs
SNARE	soluble *N*-ethylmaleimide-sensitive factor attachment protein receptor
SQSTM1	sequestosome 1
SWCNTs	single-walled carbon nanotubes
TNBC	triple-negative breast cancer
TQ	thymoquinone
ULK	Unc-51 like autophagy activating kinase (ULK1/2)
VPS	vacuolar protein sorting
